# Nanocatalytic anti-inflammatory therapy for alleviating rheumatoid lesions with endothelial cell-derived hydrosilicene

**DOI:** 10.1186/s12951-026-04930-0

**Published:** 2026-08-15

**Authors:** Jize Dong, Weihan Yu, Moran Huang, Zhou Li, Yeheng Lu, Jiwu Chen, Zhixin Chen, Xingyu Zhang

**Affiliations:** 1 Department of Plastic and Reconstructive Surgery, Shanghai Geriatric Medical Center, Shanghai, 201104 China; 2 Department of Plastic and Reconstructive Surgery, Zhongshan Hospital, Fudan University, Shanghai, 200032 China; 3 Department of Sports Medicine, Shanghai General Hospital, Shanghai Jiao Tong University School of Medicine, Shanghai, 200080 China; 4 Shanghai Frontiers Science Center of Nanocatalytic Medicine, Shanghai Tenth People’s Hospital, School of Medicine, Tongji University, Shanghai, 200331 China; 5 Department of Orthopaedics, Shanghai Sixth People’s Hospital, Shanghai Jiao Tong University School of Medicine, Shanghai, 200233 China

**Keywords:** Biocatalytic nanosheets, Endothelial cell membrane, Neutrophil, Reactive oxygen species, NETs suppression

## Abstract

Severe inflammatory damage of soft tissues and progressive bone destruction are hallmark features of rheumatoid arthritis (RA), in which over-activated neutrophils and excessive neutrophil extracellular traps (NETs) play a pivotal role during the early stages of the disease. Conventional drug therapies, such as methotrexate (MTX), are often insufficient in clinical practice, highlighting the urgent need for more targeted and definitive interventions at disease onset. Recently, accumulating evidences indicate that reactive oxygen species (ROS) act as a key trigger for NETs formation. In this study, we developed a biomimetic nanocatalytic system by coating hydrosilicene (HSI) nanosheets with natural endothelial cell membranes (HSI@ECM). This bio-inspired design mimicked typical endothelial-neutrophil adhesion, enabling targeted delivery of the nanosystem to over-activated neutrophils at the inflammatory sites. The fabricated HSI@ECM subsequently released hydrogen gas in situ and selectively scavenged excessive ROS within the neutrophils, thereby inhibiting NETs formation and preserving mitochondrial function. In vivo experiments in a collagen-induced arthritis (CIA) mouse model demonstrated that HSI@ECM significantly alleviated joint swelling, inflammatory cell infiltration and cartilage-bone damage, outperforming both the uncoated nanosheets and MTX. Overall, this work presents a nanocatalytic anti-inflammatory therapeutic strategy with promising potential for mitigating rheumatoid soft tissue, cartilage and bone damage.

**Graphical Abstract:**

**Figure:**
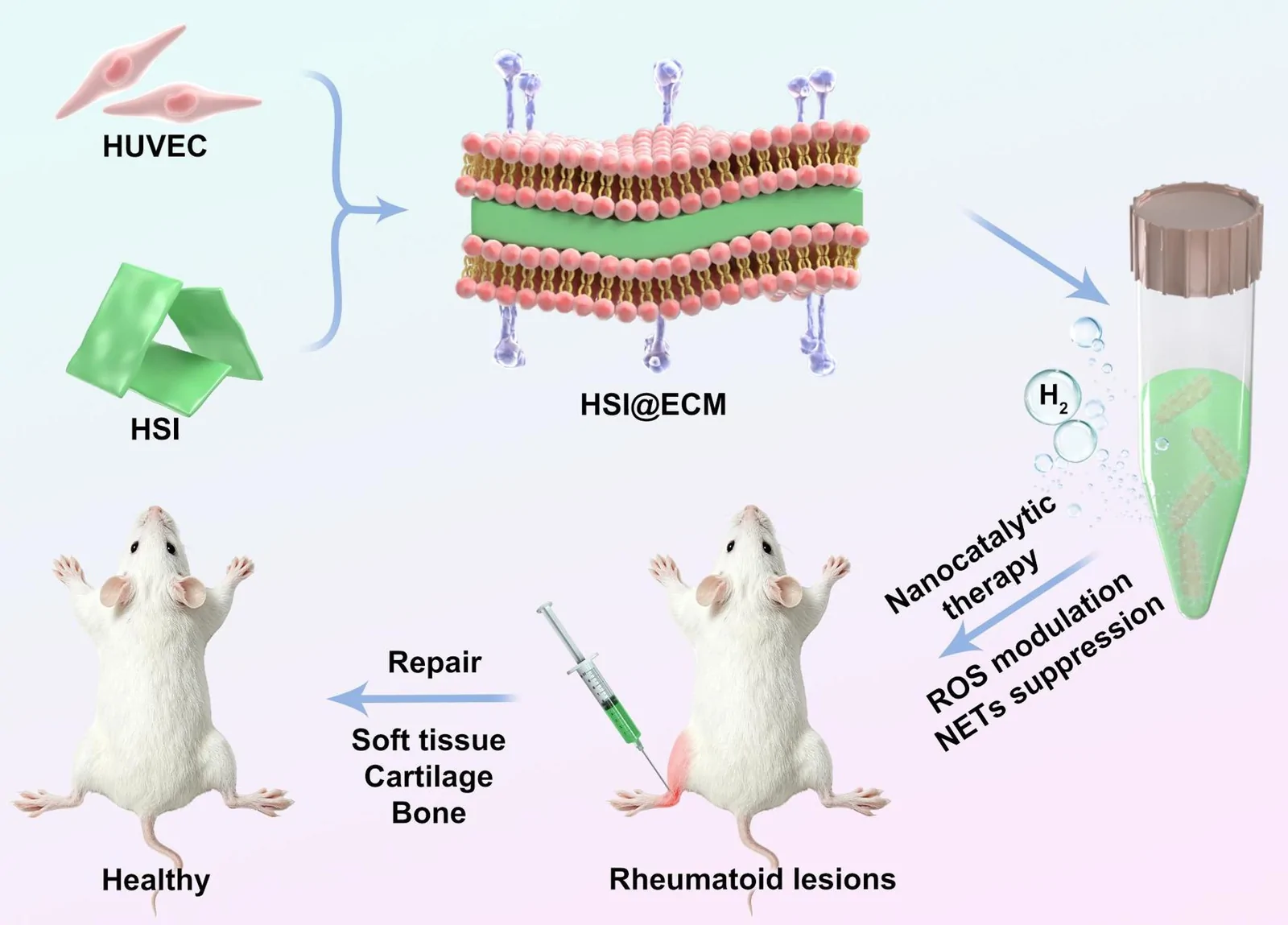

**Supplementary Information:**

The online version contains supplementary material available at https://doi.org/10.1186/s12951-026-04930-0.

## Introduction

The inflammatory infiltration of soft tissues as well as the progressive destruction of articular cartilage and bone driven by systemic immune pathology commonly exist in rheumatoid arthritis (RA). They are leading to severe disability and substantial socioeconomic burden worldwide, affecting approximately 1% of the population [1, 2]. Classical features of RA include extensive immune cell infiltration in soft tissues, synovial hyperplasia and evolving joint deformity [3]. Many current therapeutic options, such as disease-modifying antirheumatic drugs (DMARDs) and corticosteroids, could partially control disease progression. However, their clinical efficacy is often limited by adverse effects and high relapse rates after withdrawal, emphasizing the urgent need for more effective and durable treatment strategies [4].

RA is typically governed by a complex immune-inflammatory network. As the earliest innate immune cells infiltrating the synovial tissues, over-activated neutrophils play a central effector role [5, 6]. Traditionally, neutrophils are considered to mediate anti-bacterial process and tissue damage through the release of cytotoxic mediators, including reactive oxidants and proteolytic enzymes. Recently, more evidences have identified neutrophil extracellular traps (NETs) as a critical link between innate and adaptive immunity, thereby amplifying the pathogenic cascades in RA [7, 8].

The formation of NETs contributes to joint damage in RA. Firstly, NETs provide a rich source of citrullinated histone H3 (CitH3) generated by peptidylarginine deiminase 4 (PAD4), promoting the breakdown of immune tolerance [9, 10]. Secondly, excessive NETs activate fibroblast-like synoviocytes, induce pro-inflammatory transformation and enhance antigen presentation to T cells, further bridging innate and adaptive immune responses [11, 12]. Thirdly, myeloperoxidase (MPO) and neutrophil elastase (NE) embedded within NETs directly degrade cartilage and bone matrix components, together exacerbating joint destruction [13]. Therefore, these studies position NETs as a promising therapeutic target for RA intervention.

Furthermore, increasing evidences suggest that reactive oxygen species (ROS) play an important role in driving the production of NETs [14, 15]. Neutrophils from RA patients exhibit elevated ROS production, accompanied by increased oxidative stress markers in synovial fluids [16, 17]. Moreover, intracellular ROS could activate PAD4, leading to histone citrullination and subsequent NETs formation [18]. Hence, modulation of ROS represents a rational strategy to suppress aberrant NETs. However, given that ROS also serve essential physiological functions, non-specific anti-inflammatory approaches may disrupt normal redox homeostasis. This underscores the necessity for a targeted and precisely controlled anti-inflammatory platform.

In recent years, gas-based nanocatalytic systems have emerged as an applicable approach for redox regulation [19, 20]. Among them, two-dimensional hydrosilicene (HSI) nanosheets possess a unique capacity to store hydrogen atoms and sustainably release hydrogen (H_2_), thereby selectively neutralizing cytotoxic free radicals under pathological oxidative stress conditions [21, 22]. Nevertheless, in vivo application of HSI nanosheets is limited by rapid clearance and insufficient targeting to inflamed tissues. To overcome these limitations, cell membrane-coating technology has been developed, enabling the nanosystems to inherit the biological functionalities of source cells, including prolonged circulation, immune evasion and enhanced targeting capability [23–25]. Therefore, cell membrane functionalization represents a potential strategy to improve the therapeutic performance of HSI nanosheets for ROS modulation.

**Scheme 1:**
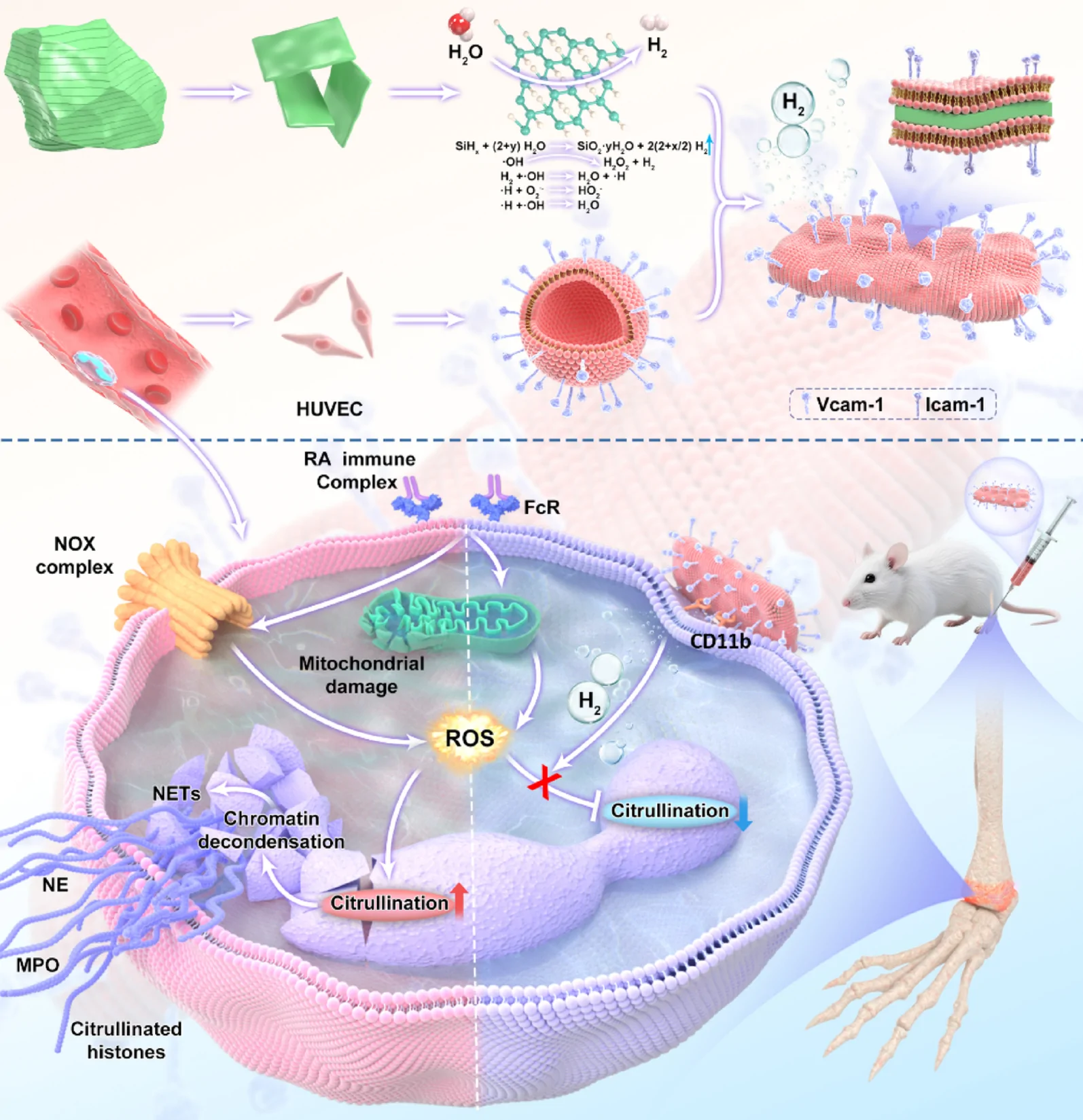
The schematic illustration of the preparation and functional mechanism of the nanocatalytic HSI@ECM. HSI@ECM was fabricated by coating HSI nanosheets with HUVEC membranes, and the H_2_ generation relied on the reaction: SiH_x_ + (2 + y) H_2_O → SiO_2_·yH_2_O + 2(2 + x/2) H_2_↑. Upon injection into the mouse footpad, HSI@ECM selectively targeted and adhered to the activated neutrophils via potential CD11b-ICAM-1/VCAM-1 binding. This targeted interaction enabled sustained H_2_ release, further effectively scavenging excessive ROS, protecting mitochondrial function and suppressing NETs in situ, thereby mitigating the pathogenic inflammatory response in RA

Herein, we report a biomimetic nanocatalytic system constructed by coating HSI nanosheets with human umbilical vein endothelial cell (HUVEC) membranes (HSI@ECM). This bio-inspired design exploits the natural adhesion interactions between endothelial cells and neutrophils, specifically through the binding of endothelial adhesion molecules of ICAM-1 and VCAM-1 to their corresponding ligands (e.g., CD11b) on neutrophils [26–28]. By mimicking this physiological adhesion process, HSI@ECM could selectively anchor to the over-activated neutrophils and hitchhike to the inflamed joint sites. Once localized, HSI@ECM enables sustained H_2_ release in situ, efficiently scavenges excessive intracellular ROS, and consequently inhibits NETs formation at its source during RA treatment (Scheme 1).

## Materials and methods

### Synthesis of HSI nanosheets

HSI nanosheets were synthesized by modifying a previously reported method [21]. Briefly, 1 g of Zintl phase CaSi_2_ precursor was added to 100 mL of pre-cooled concentrated hydrochloric acid (−25 °C) and reacted under magnetic stirring (500 rpm) for 7 days. The resulting pale green product was collected by centrifugation (10,000 g, 10 min) and washed repeatedly with ethanol. To reduce the lateral size, the product was dispersed in 100 mL ethanol and subjected to probe sonication for 24 h in an ice bath. The suspension was then centrifuged (15,000 g, 10 min) to obtain smaller-size HSI nanosheets.

### Characterization of HSI nanosheets

The surface morphology of CaSi_2_ and HSI nanosheets was examined using scanning electron microscopy (SEM, ZEISS Sigma 300, Germany). The microstructure and elemental composition of HSI nanosheets were analyzed by transmission electron microscopy (TEM, FEI TF20, USA) equipped with the energy-dispersive X-ray spectroscopy (EDS). Atomic force microscopy (AFM) measurements were conducted using a Dimension ICON system (Bruker, Germany). Fourier transform infrared (FT-IR) spectra were recorded on a PerkinElmer Frontier spectrometer (USA). Raman spectroscopy (DXR3 SmartRaman, Thermo Fisher Scientific, USA) was employed to evaluate structural changes in HSI nanosheets before and after reaction with H_2_O_2_ solution.

### Cell membrane extraction

Endothelial cell membranes and red blood cell (RBC) membranes were extracted following previously reported protocols [24, 25]. For endothelial cell membranes, HUVECs were incubated in hypotonic lysis buffer (20 mM Tris-HCl, 10 mM KCl, 2 mM MgCl_2_, pH 7.4, supplemented with protease inhibitors) on ice for 30 min. Cells were then disrupted using a probe sonicator (150 W, 3 min) on ice. The homogenate was centrifuged at 8,500 g for 10 min to remove debris and organelles. The supernatant was further ultracentrifuged at 140,000 g for 30 min to pellet the membranes. The pellet was washed once with washing buffer (10 mM Tris-HCl, 1 mM EDTA, pH 7.5). For fluorescence labeling, HUVECs were pre-incubated with DiD (80 µg/mL) for 1 h prior to membrane extraction.

To obtain RBC membranes, pre-washed RBCs were resuspended in 0.25×PBS and incubated on ice for 20 min, followed by centrifugation (800 g, 5 min). The supernatant containing hemoglobin was discarded, and the pellet was repeatedly processed until hemoglobin was completely removed. The purified cell membranes were resuspended in water at a protein concentration of 4 mg/mL and stored at −80 °C.

### Synthesis of HSI@ECM

HSI@ECM was prepared through an ultrasound-assisted membrane fusion method. HUVEC membranes (quantified by BCA assay at 4 mg/mL) were mixed with HSI nanosheets at a weight ratio of 1:2 in PBS. The mixture was co-sonicated using a bath sonicator (Fisher Scientific FS30D, USA) for 3 min. The resulting HSI@ECM was purified by ultracentrifugation (100,000 g, 1 h, 4 °C) and resuspended in PBS. As a control, RBC membrane-coated nanosheets (HSI@RBCM) were prepared using the same procedure. The mass of the products was calculated based on the content of HSI nanosheets.

### Characterization of HSI@ECM

The morphology and elemental distribution of HSI@ECM were analyzed by TEM and EDS. TEM imaging (Hitachi HT-7800, Japan) was performed after negative staining with 2% phosphotungstic acid. Hydrodynamic size and zeta potential were then measured by dynamic light scattering (DLS) using a NanoBrook 90Plus PALS system (Brookhaven, USA). Western blot (WB) analysis was conducted to verify the presence of endothelial membrane proteins (mainly ICAM-1 and VCAM-1) on HSI@ECM.

### In vitro antioxidant capacity and cytotoxicity assay

The reaction between the above product and 1mM H_2_O_2_ solution was recorded, and the real-time H_2_ gas generation was evaluated using a simple water-displacement apparatus. Hydroxyl radical scavenging activity was assessed using a commercial assay kit (Solarbio, China), and the absorbance was measured with a microplate reader (Infinite E Plex, Tecan, Switzerland). Total antioxidant capacity was evaluated using an ABTS assay kit (Beyotime, China) according to the manufacturer’s instructions.

For cellular toxicity assay, HUVECs were seeded in 96-well plates at a concentration of 5 × 10^3^ cells per well and cultured overnight. After co-culture with HSI nanosheets and HSI@ECM at concentrations of 0, 50, 100 and 200 µg/mL for durations of 24, 48, and 72 h, 100 µL of CCK-8 working solution (Beyotime, China) was added to each well and incubated in darkness at 37 °C for 2 h. Subsequently, the absorbance at 450 nm was measured using a microplate reader (Infinite E Plex, Tecan, Switzerland).

### Neutrophil isolation and cell culture

Human peripheral blood neutrophils were isolated from a healthy donor using a density gradient centrifugation kit (Solarbio, China). All procedures were approved by the Institutional Review Board of Shanghai General Hospital (Approval No. 2024KS439) and complied with the Declaration of Helsinki. Blood samples were layered into separation medium and centrifuged at 600 g for 25 min. The neutrophil layer was collected, subjected to RBC lysis, and washed twice with PBS (300 g, 5 min). Cells were resuspended in phenol red-free RPMI-1640 complete medium and cultured at 37 °C with 5% CO_2_.

### Intracellular ROS modulation

Neutrophils were seeded in 24-well plates and incubated with an ROS-sensitive fluorescent probe (Tongren, China) for 30 min. Cells were then treated with PMA (100 nM) and different concentrations of HSI nanosheets and HSI@ECM for 30 min. Fluorescence images were acquired, and the average fluorescence intensity was quantified. For kinetic analysis, cells were seeded in black 96-well plates and treated with PMA along with HSI nanosheets, HSI@ECM and a classic antioxidative drug N-acetylcysteine (NAC, MCE, USA) of 10 mM. ROS levels were continuously monitored using a fluorescence microplate reader.

### Cellular adhesion assay

The content of CD11b protein was detected by fluorescence in the activated neutrophils and then the neutrophils were co-incubated with HSI@ECM for 4 h, and washed twice with PBS. Cells were then fixed with 2.5% glutaraldehyde (0.1 M PBS, pH 7.4) at 4 °C for 2 h, followed by dehydration through a graded ethanol series. Then, the products were critical-point dried, sputter-coated with platinum, and observed using SEM (ZEISS Gemini SEM 300, Germany).

### NETs formation analysis

Activated neutrophils were firstly labeled with the membrane dye DiD (5 µM) at 37 °C in the dark for 15 min. After washing, the cells were seeded onto poly-L-lysine-coated confocal dishes and treated according to the following groups: PMA (100 nM), HSI nanosheets (100 µg/mL), HSI@ECM (100 µg/mL) and NAC (10 mM). To visualize NETs, the cell-impermeable nucleic acid dye SYTOX Green (5 µM) was added and incubated in the dark for 10 min, followed by counterstaining with Hoechst dye (5 µg/mL) for 10 min. NETs was immediately observed using an inverted fluorescence microscope. For further detailed analysis of NETs components, neutrophils were fixed with 4% paraformaldehyde, permeabilized with 0.1% Triton X-100 and blocked with 5% BSA. Samples were incubated with primary antibodies (including CitH3, MPO, NE and PAD4 proteins) at 4 °C overnight, followed by the corresponding fluorescently labeled secondary antibodies. The nuclei were counterstained with Hoechst dye, and images were acquired using a fluorescence microscope.

### Mitochondrial ultrastructure and membrane potential

For ultrastructural analysis, neutrophils were fixed with 2.5% glutaraldehyde and post-fixed with 1% osmium tetroxide. After dehydration and embedding, ultrathin sections were examined by TEM. Mitochondrial membrane potential was evaluated using a JC-1 assay kit (Beyotime, China). Cells were treated with PMA for 4 h, stained with JC-1 dye and analyzed by fluorescence microscopy. The cellular viability and stability of neutrophils were reflected by the mitochondrial functions.

### CIA model and therapeutic intervention

All animal experiments were approved by the Animal Experiment Ethics Committee of Shanghai General Hospital (Approval No. 2024SC030) and conducted according to the National Institutes of Health Guide for the Care and Use of Laboratory Animals. Male DBA/1 mice (6–8 weeks old) were housed under specific pathogen-free conditions. The CIA model was established as previously described [29]. Briefly, the mice were anesthetized and immunized via intradermal injection at the tail base with an emulsion containing 100 µg bovine type II collagen (Chondrex, 20022, USA) mixed with an equal volume of Complete Freund’s Adjuvant (Chondrex, 7001, USA). A booster immunization was administered using collagen emulsified in Incomplete Freund’s Adjuvant (Chondrex, 7002, USA) on day 21. At the first 28 days, the mice were randomly assigned into five groups and received injections into hind footpads every other day: control, PBS, HSI nanosheets (100 µg/mL, 10 µL per paw), HSI@ECM (100 µg/mL, 10 µL per paw) and MTX (5 mg/kg). Arthritis severity was scored weekly for each paw on a scale of 0–4 each item [29], resulting in a maximum possible score of 16 per mouse.

### Micro-CT analysis

Joint samples were scanned using a high-resolution micro-CT system (SkyScan 1176, Bruker, Belgium) under the following settings: 65 kV voltage, 385 µA current and 520 ms exposure time. The projection images were reconstructed into three-dimensional volumes using CTvox software (v3.3.0, Bruker), and morphometric parameters were quantified from the 3D models using CTan software (v1.18.4.0, Bruker).

### Histological staining

Joint tissues were fixed, decalcified, paraffin-embedded and sectioned. Histological evaluation was performed using the following stains, which were Hematoxylin and Eosin (HE): hematoxylin working solution and 1% Eosin Y; Toluidine Blue (TB): Weigert’s hematoxylin solution followed by Toluidine Blue solution; Safranin O/Fast Green (SO/FG): Weigert’s hematoxylin, Fast Green solution and 0.1% Safranin O solution. All stained sections were observed under a light microscope. The Mankin’s scoring system was utilized for quantitative analysis.

### Immunofluorescence staining

For the early-stage immunofluorescence detection of NETs in vivo, CIA mice were sacrificed 4 weeks after first immunization. Ankle joint tissues were harvested, fixed, decalcified, paraffin-embedded and then sectioned. Tissue sections underwent antigen retrieval, endogenous peroxidase inactivation and blocking. Sections were then incubated with primary antibodies at 4 °C overnight, followed by fluorescently-labeled secondary antibodies. Nuclei were counterstained with DAPI dye, and images were captured using an inverted fluorescence microscope.

### Statistical analysis

All data are presented as mean ± standard deviation (SD). One-way analysis of variance (ANOVA) was performed using GraphPad Prism software (v8.3). A p-value < 0.05 was considered statistically significant (ns represented no significant difference, *p < 0.05, **p < 0.01, ***p < 0.001, ****p < 0.0001).

## Results and discussion

### Synthesis and characterization of HSI nanosheets

**Fig. 1:**
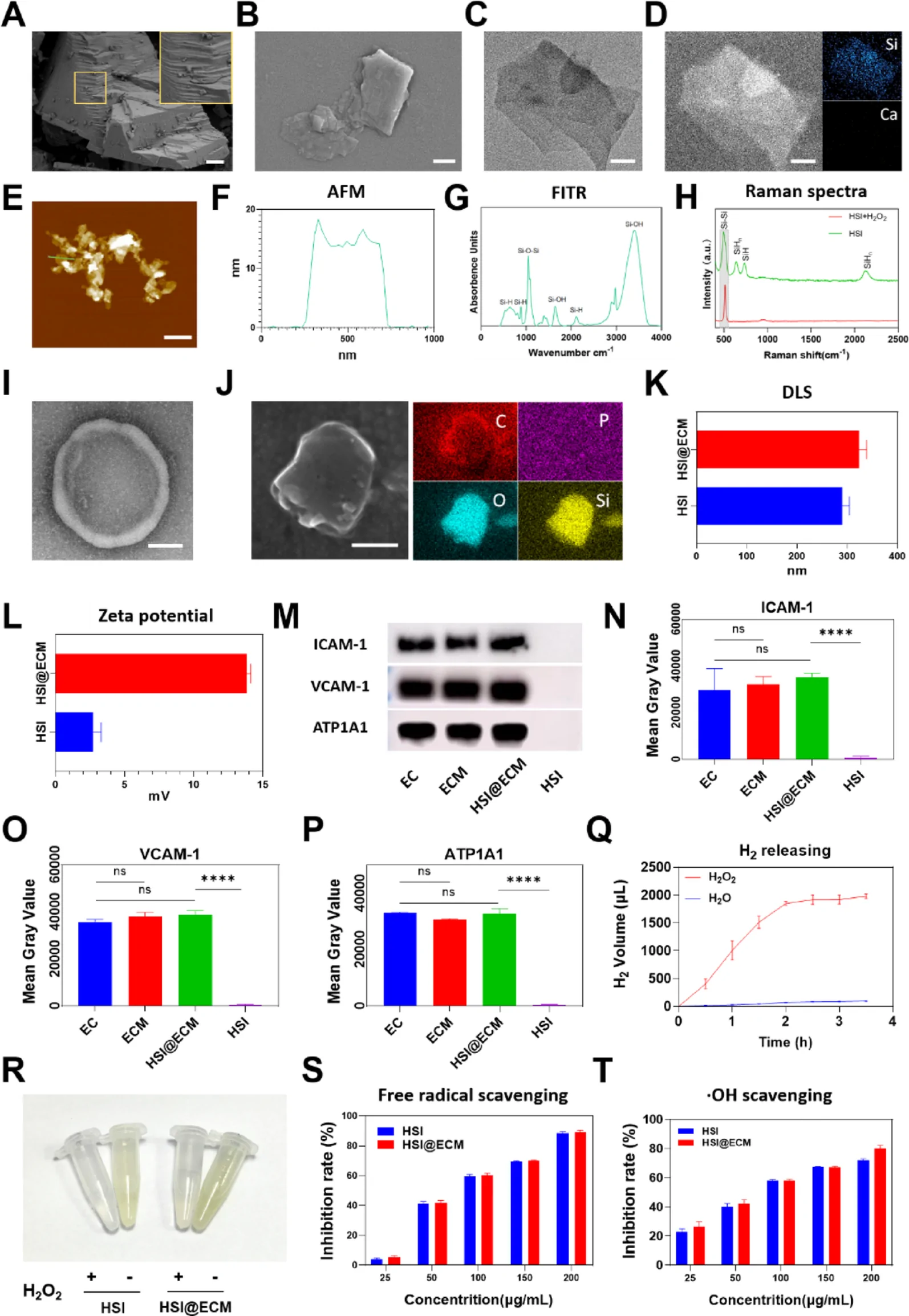
Characterization of HSI nanosheets and HSI@ECM. (**A**) SEM image of CaSi_2_ (scale bar: 5 μm). (**B**) SEM image of HSI nanosheets (scale bar: 200 nm). (**C**) TEM image of HSI nanosheets (scale bar: 100 nm). (**D**) EDS elemental mapping of HSI nanosheets (scale bar: 100 nm). (**E**,** F**) AFM image and measurement of HSI nanosheets (scale bar: 1 μm). (**G**) FT-IR spectrum of HSI nanosheets. (**H**) Raman spectra of HSI nanosheets and its corresponding reaction product after mixing with H_2_O_2_ solution. (**I**) TEM image of endothelial cell (EC) membrane (ECM)-derived vesicle (scale bar: 100 nm). (**J**) SEM image and EDS mapping of HSI@ECM (scale bar: 500 nm). (**K**) Hydrodynamic diameters measured by DLS analysis (*n* = 3). (**L**) Zeta potentials of HSI nanosheets and HSI@ECM (*n* = 3). (**M**) Representative WB analysis showing characteristic protein (ICAM-1 and VCAM-1) bands of EC lysates, ECM and HSI@ECM. ATP1A1 protein served as the internal reference. (**N-P**) Quantitative analysis of the protein bands of WB results (*n* = 3, ns represented no significant difference, ****p < 0.0001). (**Q**,** R**) The real-time quantitative volume of H_2_ gas generation from HSI@ECM and the reaction with H_2_O_2_ solution. (**S**,** T**) Free radical and hydroxyl radical (·OH) scavenging capacities of the products (*n* = 3)

HSI nanosheets modified with Si-H bonds were synthesized via a one-step wet chemical exfoliation method. Specifically, free-standing monolayer silicene was obtained from the layered Zintl phase precursor calcium silicide (CaSi_2_). SEM image showed the typical stacked microstructure of CaSi_2_ (Fig. 1A). Through a topochemical deintercalation process, Ca^2+^ ions were selectively removed from the silicide lattice upon treatment with concentrated hydrochloric acid according to the reaction: 3CaSi_2_ + 6HCl → 3CaCl_2_ + 6 H-Si [21]. The low-temperature exfoliation process generated two-dimensional silicon nanosheets with surfaces covalently bonded to abundant hydrogen atoms via the Si-H bonds. Subsequent probe sonication further reduced the lateral dimensions of the nanosheets via cavitation effects, further making them more suitable for biomedical applications. SEM and TEM observations confirmed that the resulting nanosheets exhibited a reduced lateral size of approximately 300 nm (Fig. 1B, C).

Furthermore, elemental mapping by EDS demonstrated the distribution of silicon with an obviously decreased calcium signal, confirming the effective removal of calcium during acid treatment (Fig. 1D). AFM analysis further revealed that the thickness of the HSI nanosheets was less than 15 nm (Fig. 1E and F). Moreover, FT-IR spectra displayed characteristic absorption peaks at 644.1, 873.6 and 2111.7 cm^− 1^, corresponding to the rocking, bending and stretching vibrations of Si-H bonds, respectively [22]. Additional peaks at 1058.7 and 3454.3 cm^− 1^ were assigned to Si-O-Si and Si-OH groups, indicating slight surface oxidation, with non-toxic Si(OH)ₓ as the primary degradation product (Fig. 1G). Raman spectroscopy further confirmed the presence of Si-Si and Si-H vibrational modes in the pristine HSI nanosheets, whereas the characteristic Si-H peak disappeared after reaction with H_2_O_2_, suggesting consumption of Si-H bonds (Fig. 1H). On this basis, the oxidation-reductive reaction was established through hydrosilicene-dependent nanocatalytic therapy.

### Synthesis and characterization of HSI@ECM

To construct the biomimetic nanocatalytic system, cell membrane derived from HUVECs was extracted and subsequently coated onto HSI nanosheets. TEM imaging showed that the isolated HUVEC membrane vesicle exhibited a typical spherical hollow structure (Fig. 1I). Following membrane coating, SEM images revealed the presence of a membrane-like layer on the surface of HSI nanosheets, while EDS analysis indicated elemental compositions mainly consisting of C, O, P and Si (Fig. 1J).

DLS analysis further showed that the hydrodynamic diameter of HSI@ECM increased by approximately 30 nm compared to bare HSI nanosheets, accompanied by a more negative zeta potential (Fig. 1K and L). Moreover, WB experiments revealed the presence and enrichment of key endothelial cell membrane proteins in both HUVEC membrane vesicles and HSI@ECM, confirming the effective transfer of membrane components onto HSI nanosheets (Fig. 1M-P).

Subsequently, the antioxidant properties of HSI nanosheets and HSI@ECM were evaluated in vitro. The final product HSI@ECM effectively produced H_2_ gas and reacted with H_2_O_2_ solution (Fig. 1Q, R and Fig. S1). In addition, both of them exhibited strong free radical scavenging activity and hydroxyl radical elimination capacity (Fig. 1S and T). Also, the in vitro cytotoxicity results of the HSI nanosheets (Fig. S2) and HSI@ECM (Fig. S3) suggested the good cytocompatibility with HUVECs for further use. HSI@RBCM were then prepared for comparison using the same procedure and characterized (Fig. S4A and B). Taken together, the above results demonstrated the successful fabrication of HSI@ECM nanocatalytic system with preserved structural and functional properties for efficient elimination of peroxides and radicals.

### Intracellular ROS scavenging capability of HSI@ECM

**Fig. 2:**
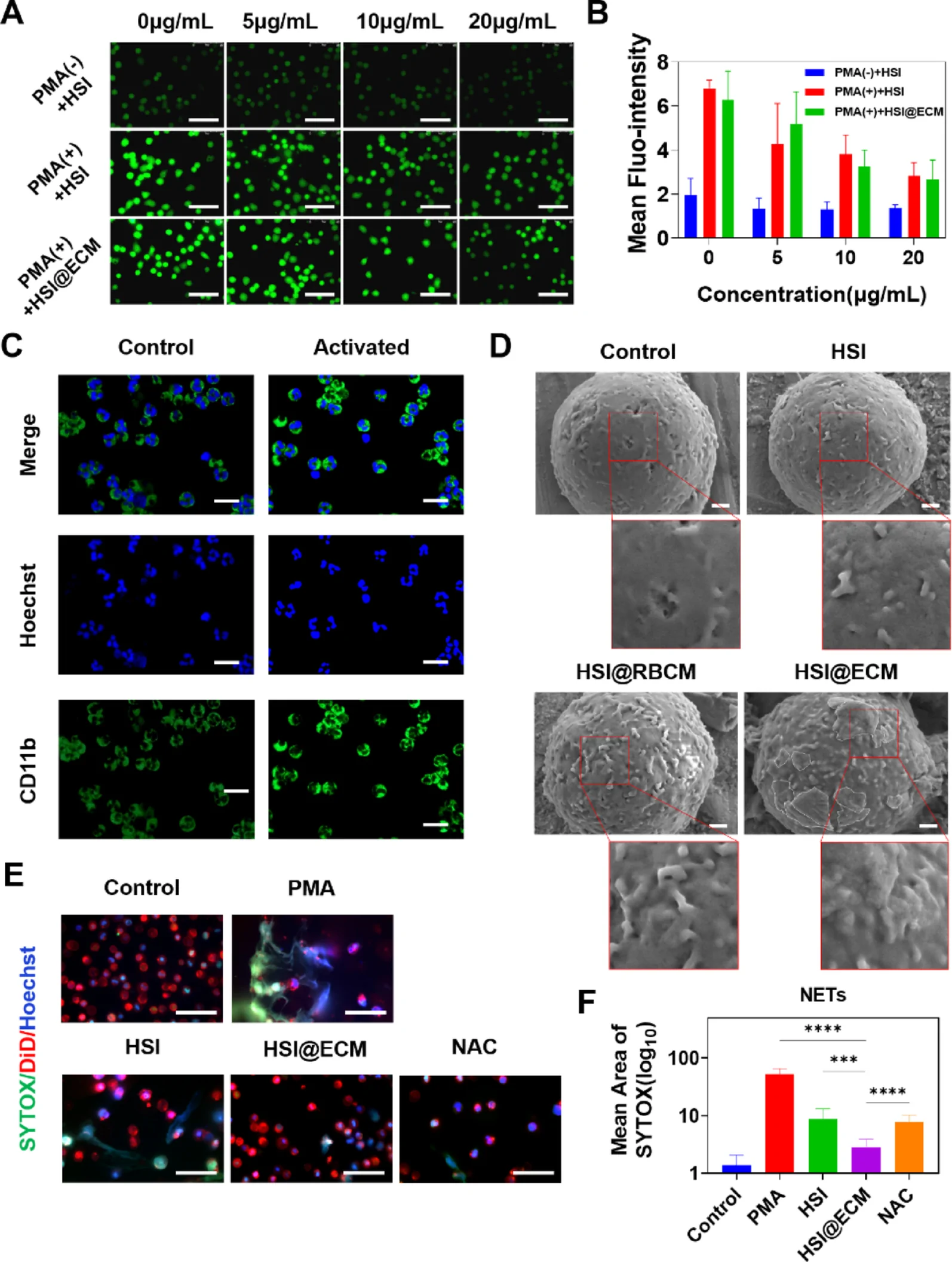
ROS scavenging capacity, adhesive property and NETs suppressive function of HSI@ECM. (**A**) Fluorescence images showing ROS scavenging by HSI nanosheets and HSI@ECM at varying concentrations (scale bars: 50 μm). (**B**) Quantitative analysis of the ROS fluorescence intensity of HSI nanosheets and HSI@ECM (*n* = 3). (**C**) The expression of CD11b protein before and after the activation of neutrophils (scale bar: 25 μm). (**D**) SEM images of neutrophil surfaces co-cultured with adhesive HSI nanosheets, HSI@RBCM and HSI@ECM (scale bars: 500 nm). (**E**) NETs formation in neutrophils illustrated by SYTOX Green/DiD staining (scale bars: 50 μm). (**F**) Quantitative analysis of fluorescence intensity of SYTOX Green (*n* = 3, ***p < 0.001, ****p < 0.0001)

Human peripheral blood neutrophils were isolated from a healthy volunteer. Wright-Giemsa staining confirmed the typical horseshoe-shaped nuclei of neutrophils (Fig. S5). To investigate the regulatory effects of HSI@ECM on neutrophil function, the isolated neutrophils were activated using PMA in vitro, followed by treatment with HSI nanosheets and HSI@ECM. Both HSI nanosheets and HSI@ECM exhibited ROS scavenging activities, with increasing efficiency at higher concentrations (Fig. 2A and B). Notably, analysis of ROS scavenging kinetic curves revealed that neither HSI nanosheets nor HSI@ECM affected physiological ROS levels (Fig. S6 and S7), whereas both of them effectively suppressed the excessive ROS (Fig. S8). These findings suggested that HSI@ECM selectively eliminated pathological ROS while preserving basal redox homeostasis, highlighting its potential as a useful nanocatalytic strategy for regulating neutrophil overactivation.

### Adhesive property of HSI@ECM

Neutrophils physiologically adhere to vascular endothelial cells via receptor-mediated interactions in response to specific chemotactic signals (e.g., previously-reported CD11b-ICAM-1/VCAM-1 binding mechanism), followed by processes such as crawling and trans-endothelial migration to reach inflammatory or injured sites [30–32]. Hence, we would evaluate whether HSI@ECM could mimic endothelial adhesion and interact with neutrophils.

In this study, the neutrophils were observed a high expression of CD11b protein after PMA-induced activation (Fig. 2C), and then incubated with HSI@ECM, with HSI nanosheets and HSI@RBCM serving as controls. Interestingly, SEM images revealed that HSI@ECM efficiently adhered to the surface of the neutrophils, whereas minimal adhesion was observed for HSI nanosheets and HSI@RBCM (Fig. 2D), implying that HSI@ECM could target the neutrophils due to activating CD11b-ICAM-1/VCAM-1 binding mechanism. Therefore, HSI@ECM possessed neutrophil-targeting capability, enabling effective cell surface adhesion for precisely biocatalyze pathological ROS.

### NETs suppression by HSI@ECM

Excessive ROS would trigger the generation of NETs. Thereafter, NETs are not merely byproducts of inflammation in RA but represent a central driver of disease initiation and progression [33]. To evaluate the influence of HSI@ECM on NETs formation, NETs were induced in human peripheral blood neutrophils using PMA in advance, followed by treatment with HSI nanosheets, HSI@ECM and NAC. Extracellular DNA release was visualized using the cell-impermeable nucleic acid dye SYTOX Green. Notably, HSI@ECM obviously suppressed NETs formation, exhibiting a significantly stronger inhibitory effect compared to both HSI nanosheets and NAC (Fig. 2E and F).

**Fig. 3:**
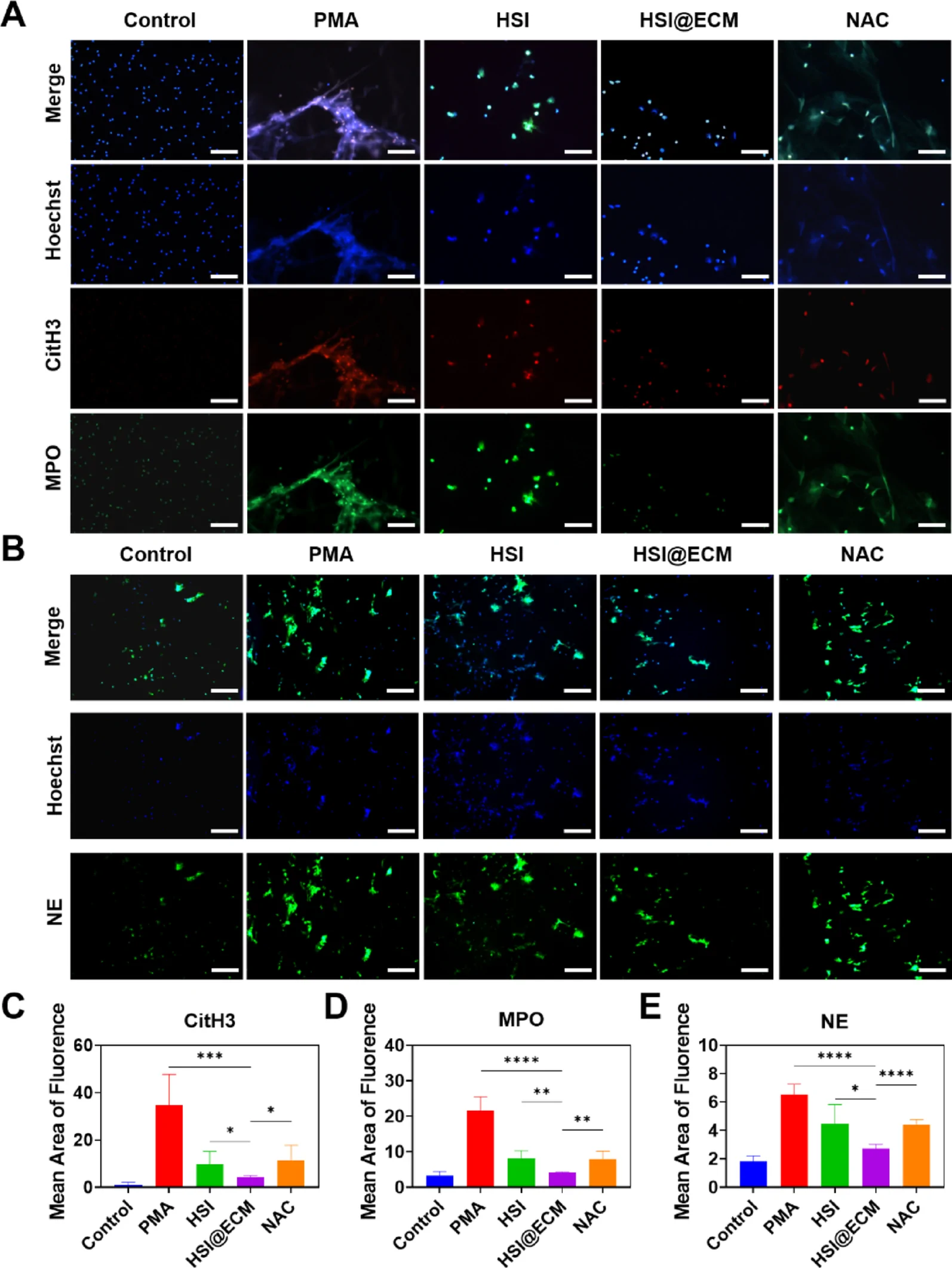
Detection of the main components of NETs in control, PMA, HSI nanosheets, HSI@ECM and NAC groups. (**A**) Immunofluorescence detection of CitH3 and MPO (scale bars: 100 μm). (**B**) Immunofluorescence detection of NE (scale bars: 200 μm). (**C-E**) Quantitative analysis of fluorescence intensities of CitH3, MPO and NE (*n* = 3, *p < 0.05, **p < 0.01, ***p < 0.001, ****p < 0.0001)

As is well known, NETs are primarily composed of CitH3, MPO and NE, which together form DNA-protein complexes responsible for their immune and pathological functions [34, 35]. To further investigate the effect of HSI@ECM on NETs components, immunofluorescence staining was performed. Consistent with the extracellular DNA results, all key NETs markers highly expressed in the PMA-stimulated group but were substantially reduced following the HSI@ECM treatment (Fig. 3A and B). Further quantitative assay also confirmed the superior NETs components suppression in HSI@ECM group (Fig. 3C-E).

Next, we further examined the PAD4 protein in these groups, aiming to explore the mechanism of NETs modulation by HSI@ECM. As shown in Fig. S9, there was an obvious elevation of PAD4 protein expression in PMA group, while the experimental groups treated with HSI nanosheets and HSI@ECM showed a diminished expression of PAD4 protein. Besides, the measured fluorescence data also indicated the clear transformative PAD4 protein from higher to lower expression after the nanocatalytic management with HSI@ECM (Fig. S10). Considering the reports by the previous studies [36, 37], the activation of PAD4 protein mainly relied on the cellular signaling of calcium ion influx, which was enhanced due to the increased cell plasma membrane permeability caused by excessive ROS. Hence the above phenomenon indicated ROS scavenging would regulate egregious NETs based on the reduction of PAD4 activity. Therefore, the nanocatalytic HSI@ECM was capable of inhibiting NETs formation at both the structural and molecular levels after ROS modulation.

### Mitochondrial protection by HSI@ECM

**Fig. 4:**
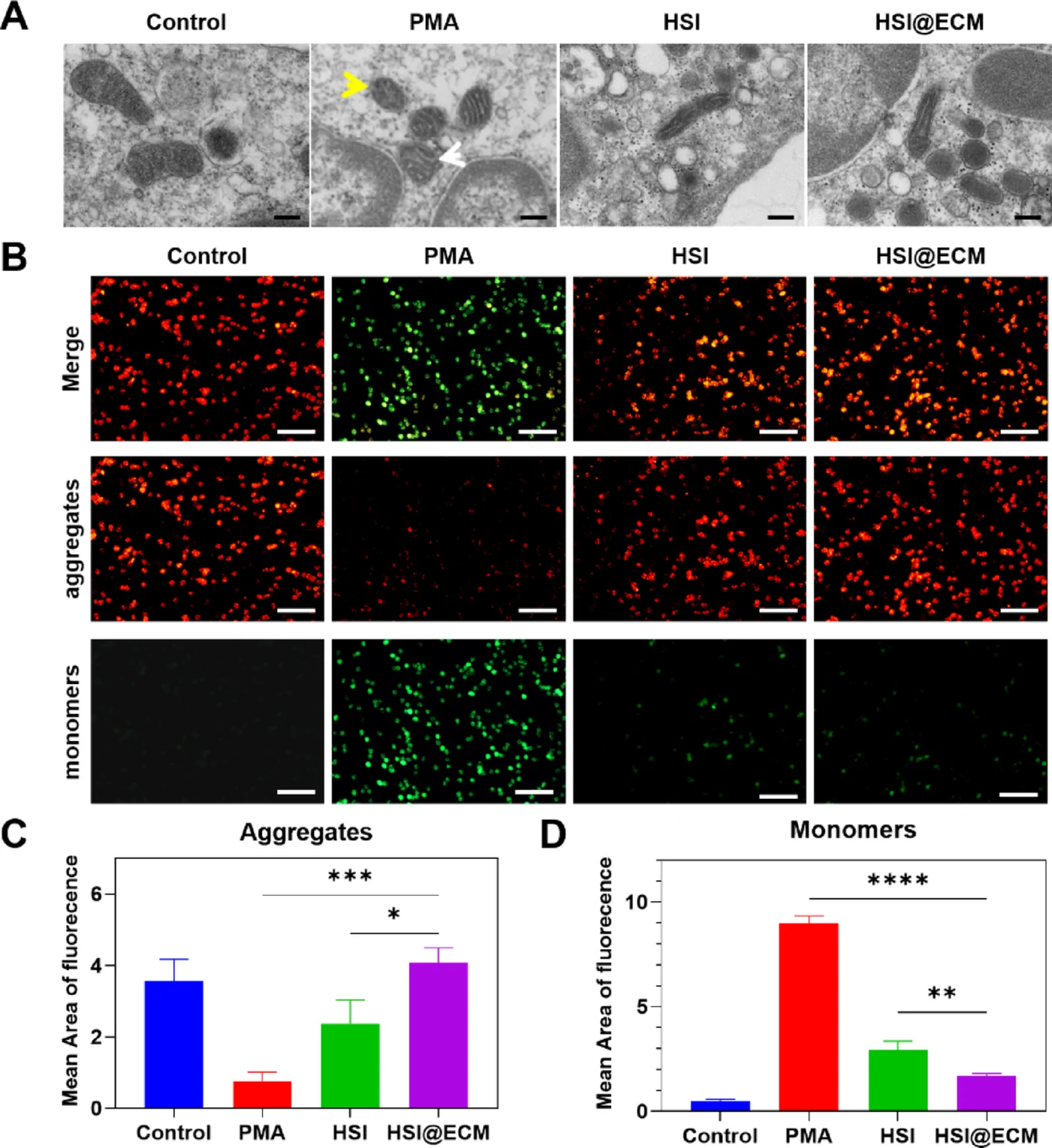
Assessment of mitochondrial protection by HSI nanosheets and HSI@ECM. (**A**) TEM images showing the ultrastructure of neutrophil mitochondria (scale bars: 250 nm). White arrow head: The mitochondrial cristae swelling. Yellow arrow head: The disruption of mitochondrial cristae. (**B**) JC-1 staining for neutrophil mitochondrial membrane potentials (scale bars: 100 μm). (**C**,** D**) Quantitative analysis of the fluorescence of the mitochondrial membrane potential illustrated in Fig. 4B (*n* = 3, *p < 0.05, **p < 0.01, ***p < 0.001, ****p < 0.0001)

Neutrophils stimulated by immune reaction would generate intracellular ROS, which in turn leads to mitochondrial damage and a reduction in mitochondrial membrane potential (Δψm) because of prolonged production of excessive ROS [38, 39]. TEM revealed that PMA stimulation induced pronounced mitochondrial cristae swelling and structural disruption, whereas these abnormalities were obviously alleviated in the presence of HSI@ECM (Fig. 4A). To further assess mitochondrial function, JC-1 staining was performed to evaluate Δψm. A higher JC-1 red/green fluorescence ratio reflects better preservation of mitochondrial membrane potential. As expected, Δψm was significantly reduced in the PMA group. In contrast, neutrophils treated with HSI@ECM exhibited a similar JC-1 red/green ratio comparable to that of the control group and significantly higher than those of the HSI nanosheets and PMA group (Fig. 4B-D). The Δψm detection results indicated that HSI@ECM apparently mitigates ROS-induced mitochondrial dysfunction, thereby preserving mitochondrial integrity and maintaining normal cell survival and function.

### Therapeutic efficacy of HSI@ECM in a CIA mouse model

**Fig. 5:**
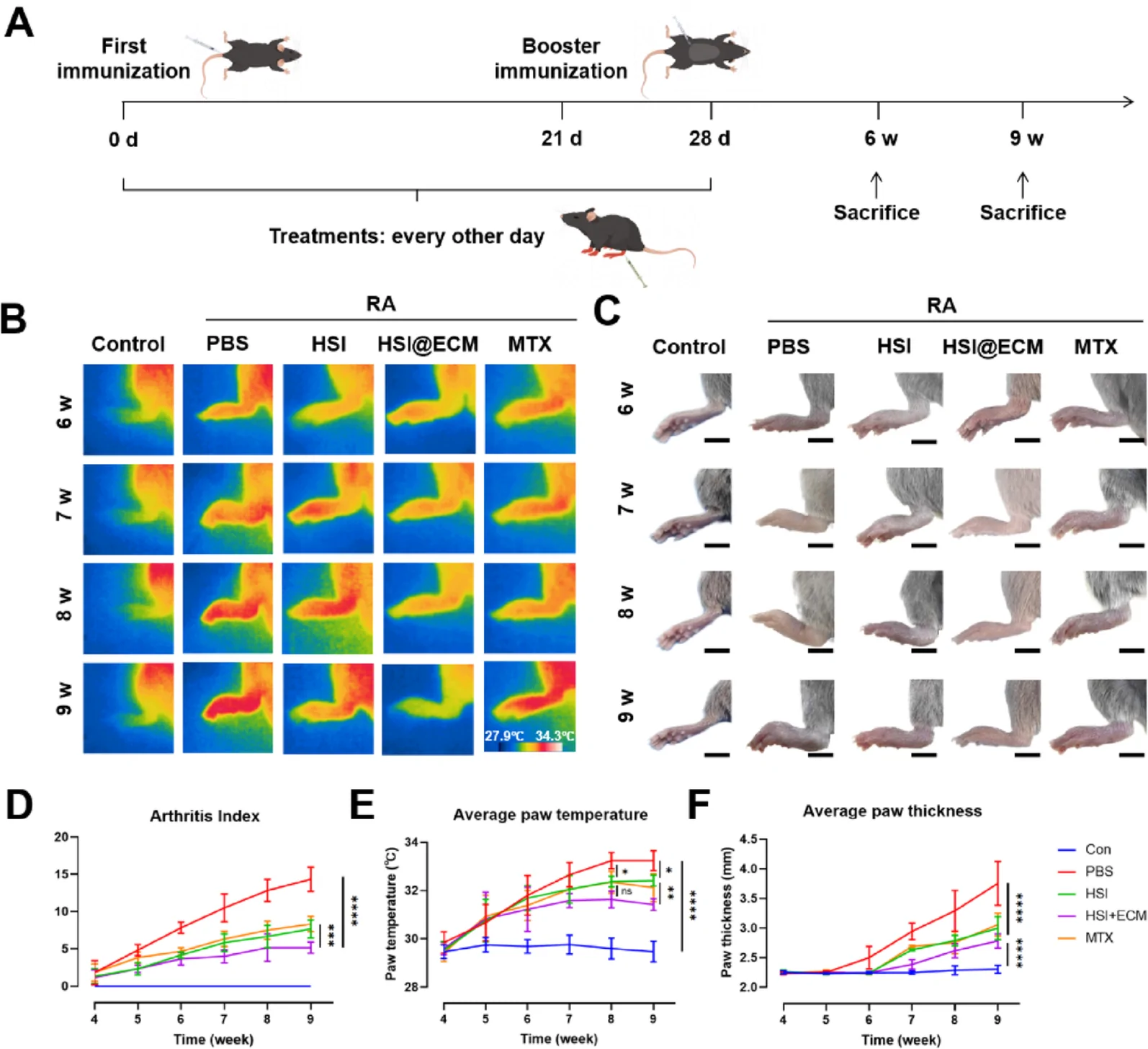
HSI@ECM palliated RA symptoms in CIA mice. (**A**) The study protocol of the therapeutic regimen with the CIA mouse model. (**B**,** C**) Thermographic images and the representative photos of left hind paws in CIA mice after different treatments (scale bars: 5 mm). (**D**) Arthritis indexes of CIA mice from week 4 to week 9 among all treatment groups (*n* = 4). (**E**,** F**) Quantitative analysis of paw temperature and thickness at different time points following treatment with HSI nanosheets, HSI@ECM and MTX, which were based on the analysis of Fig. 5B and C (*n* = 4, ns represented no significant difference, *p < 0.05, **p < 0.01, ***p < 0.001, ****p < 0.0001)

A CIA mouse model was established to evaluate the therapeutic efficacy of HSI@ECM in vivo. The mice were randomly assigned into five groups: control, PBS, HSI nanosheets, HSI@ECM and MTX. From the day of primary immunization, the corresponding treatments were administered via injections into the hind paw footpads every other day. Mice were sacrificed at 6 and 9 weeks post-immunization for further analysis (Fig. 5A). By day 28 after primary immunization, mice in PBS group began to exhibit severe local inflammation, including skin redness and soft tissue swelling, which progressively worsened over time. On the contrary, disease progression was noticeably attenuated in all treatment groups. Arthritis index scoring demonstrated that, at the experimental endpoint, all treated groups showed reduced disease severity compared to the PBS group, while the HSI@ECM group exhibiting significantly lower scores than both the HSI nanosheets and MTX groups (Fig. 5D). Consistently, quantitative measurements showed that hind paw temperature (Fig. 5B and E) and paw thickness (Fig. 5C and F) were significantly reduced in the HSI@ECM-treated mice compared to the other groups. These in vivo results implied that HSI@ECM inhibited the joint inflammation and exhibited superior therapeutic efficacy in the CIA model.

### Micro-CT analysis

**Fig. 6:**
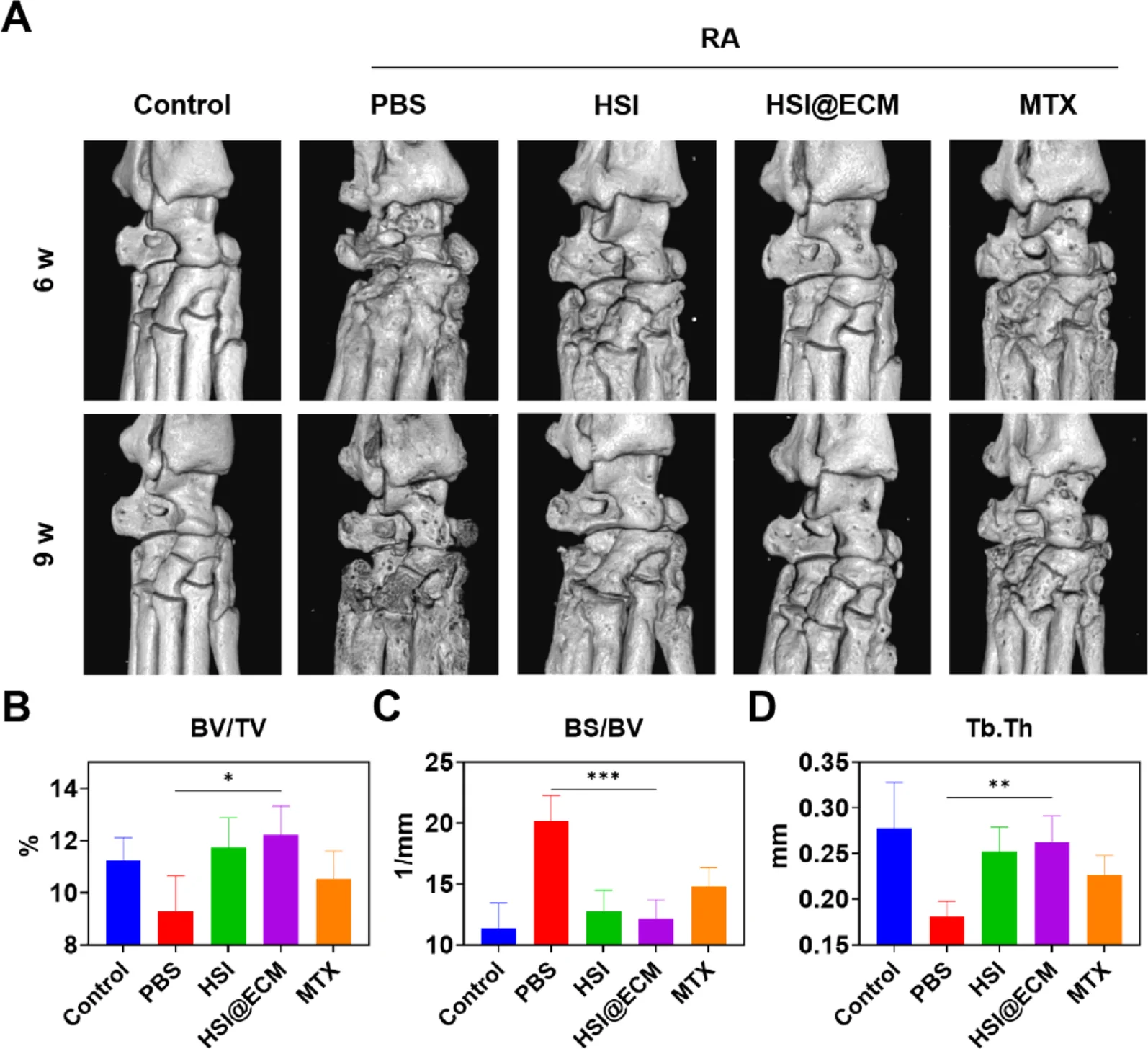
HSI@ECM alleviated bone erosion in CIA mice. (**A**) Micro-CT 3D reconstruction of the left hind paws following different treatments. (**B-D**) Quantitative analysis of the morphometric parameters based on Micro-CT results in Fig. 6A including BV/TV, BS/BV and Tb. Th values (*n* = 4, *p < 0.05, **p < 0.01, ***p < 0.001). BV/TV represented bone volume fraction (bone volume/total volume), BS/BV represented bone surface/bone volume, and Tb. Th represented the bone trabecular thickness

Micro-CT imaging was performed to evaluate bone erosion at RA lesion sites. As shown in Fig. 6A, CIA mice in PBS group exhibited severe bone erosion and irregular cortical bone surfaces. In contrast, treatment with HSI@ECM obviously inhibited bone erosion in the ankle joints, demonstrating better protective effects compared to both the HSI nanosheets and MTX groups. For quantitative assessment, key morphometric parameters, including bone volume fraction (BV/TV, Fig. 6B), bone surface fraction (BS/BV, Fig. 6C) and trabecular thickness (Tb.Th, Fig. 6D) were analyzed. PBS group showed the lowest BV/TV and Tb.Th values and the highest BS/BV value, implying the existence of severe bone destruction. These parameters were significantly improved in all treatment groups, while the HSI@ECM group exhibiting the most pronounced recovery compared to the HSI nanosheets and MTX groups.

### In vivo histological evaluation

**Fig. 7:**
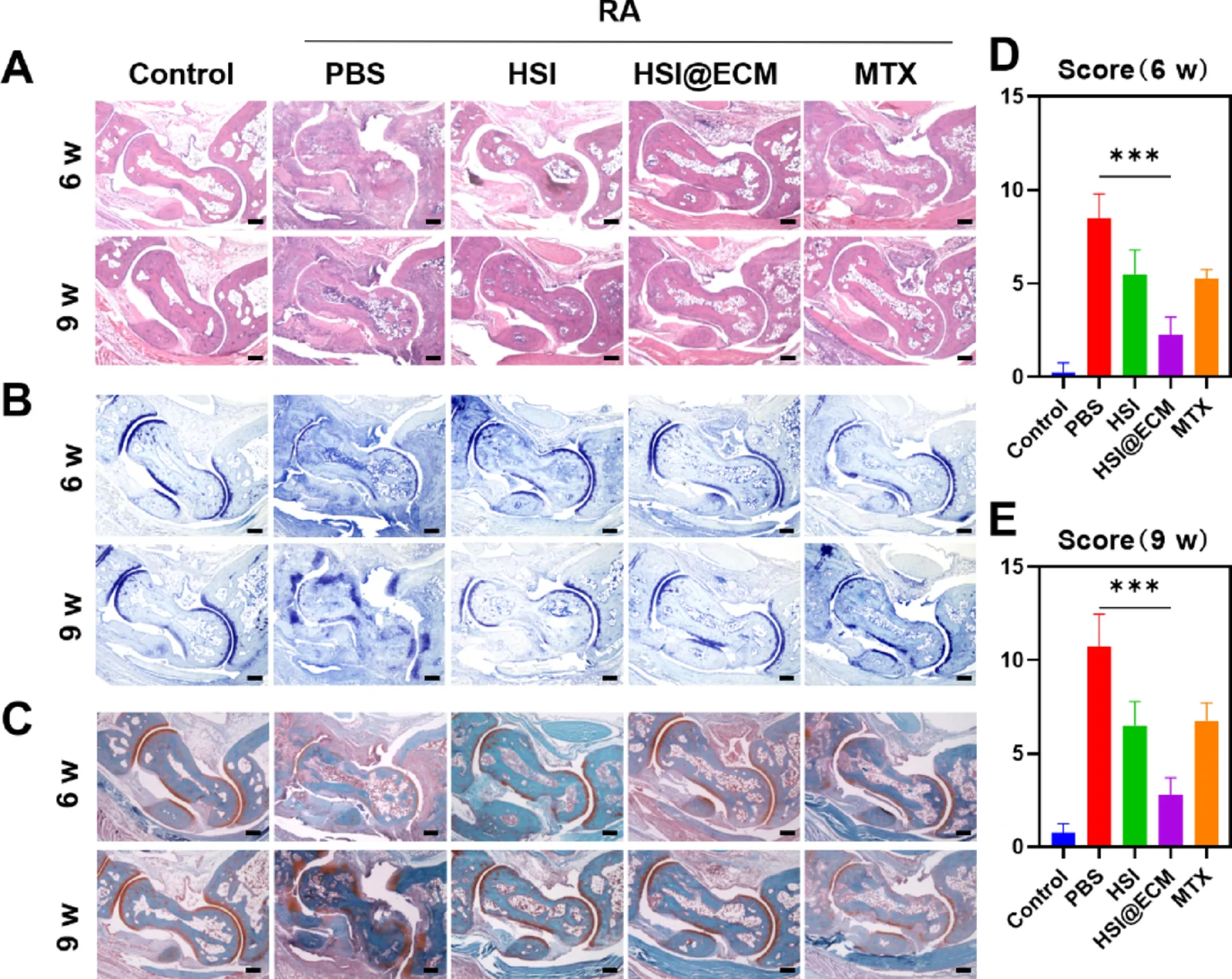
HSI@ECM alleviated soft tissue and cartilage-bone damage in CIA mice. (**A**) HE staining for histological evaluation of the hind paws. (**B**) TB staining. (**C**) SO/FG staining (all scale bars: 200 μm). (**D-E**) Mankin’s scores of cartilage structure at week 6 and week 9 post-treatment according to the SO/FG staining results in Fig. 7C (*n* = 4, ***p < 0.001). Lower value of Mankin’s score represented good quality of the tissue

Next, to further evaluate the detailed therapeutic efficacy, histological analysis was utilized on the joint tissues at 6 and 9 weeks after model establishment. HE (Fig. 7A), TB (Fig. 7B) and SO/FG (Fig. 7C) stained sections together revealed extensive inflammatory cell infiltration, synovial hyperplasia, cartilage destruction and severe bone erosion in PBS group. On the contrary, HSI@ECM obviously alleviated synovial inflammation, reduced bone erosion in the ankle joints and prevented talus destruction. Notably, articular cartilage degeneration was also significantly ameliorated, indicating both cartilage-protective and bone-preserving effects. Meanwhile, the Mankin’s scores also demonstrated that HSI@ECM significantly mitigated cartilage damage and exhibited enhanced therapeutic efficacy compared to both HSI nanosheets and MTX (Fig. 7D and E). These in vivo findings collectively confirmed the robust therapeutic potential of HSI@ECM in ameliorating soft tissue, cartilage and bone damage in RA.

### In vivo NETs inhibition by HSI@ECM

**Fig. 8:**
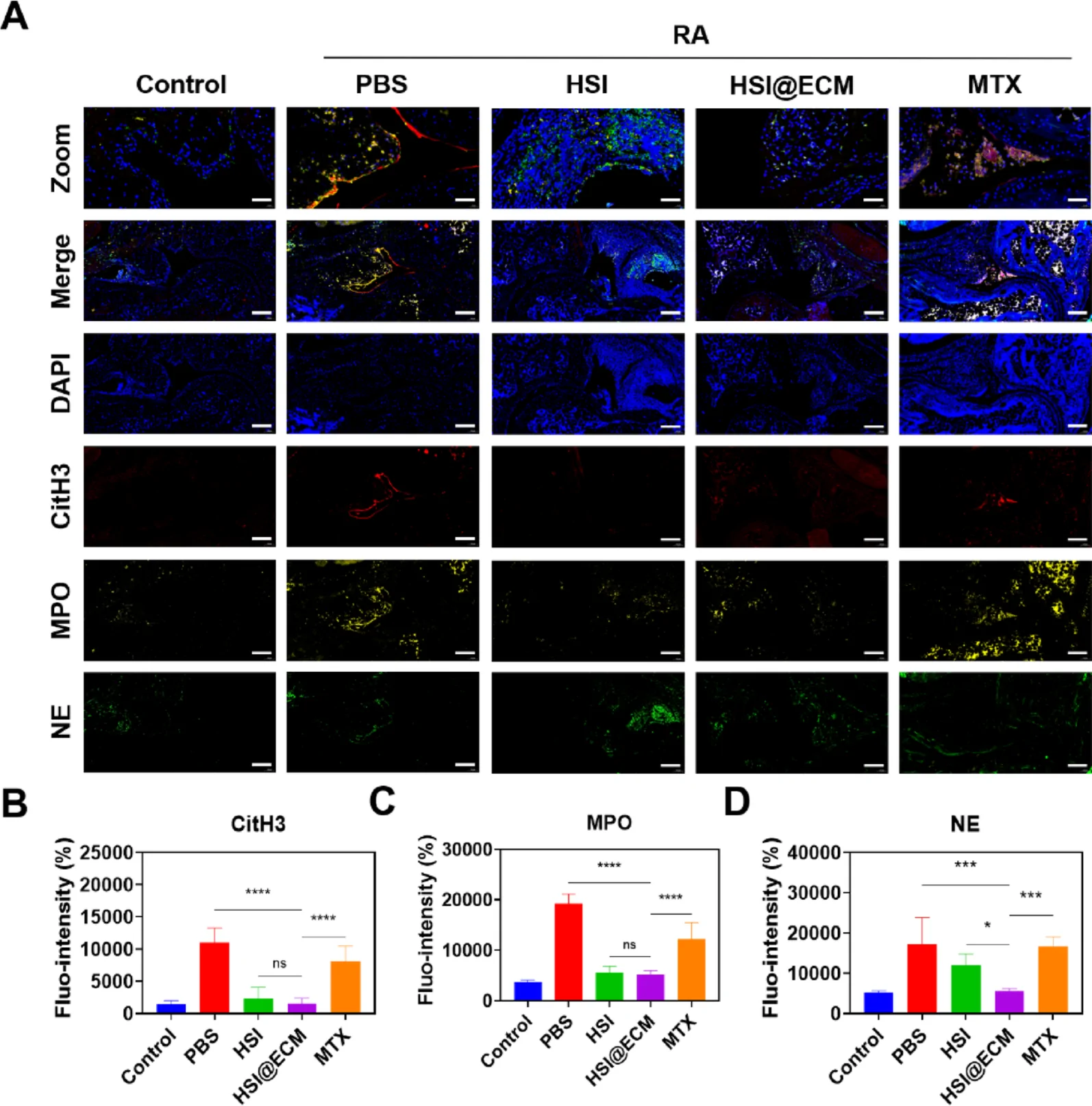
HSI@ECM suppressed NETs in vivo. (**A**) Immunofluorescent images of CitH3, MPO and NE with zoomed and merged pictures in the groups of control, PBS, HSI nanosheets, HSI@ECM and MTX (scale bars: 200 μm). (**B-D**) Quantitative analysis of CitH3, MPO and NE originated from Fig. 8A (*n* = 4, ns represented no significant difference, *p < 0.05, ***p < 0.001, ****p < 0.0001)

To study the therapeutic mechanism of HSI@ECM, a molecular immunofluorescence staining was performed in vivo to detect NETs-associated markers, including CitH3, MPO and NE, in the ankle joint tissues of CIA mice (Fig. 8A). Quantitative analysis of fluorescence intensity was subsequently conducted. The results showed a consistent trend among all NETs markers (Fig. 8B-D). In detail, CitH3, MPO and NE abundantly expressed in the PBS group, whereas their levels reduced to varying degrees in all treatment groups. Notably, HSI@ECM group exhibited the most significant reduction in these markers compared to HSI nanosheets and MTX groups.

Given these above experimental results, by utilizing the advantage of targeting the activated neutrophils, HSI@ECM achieved a nanocatalytic system for ROS-specific therapy, whose functions could depend on smaller dosage, lower off-target effect and excellent biosafety. Therefore, HSI@ECM was finally demonstrated to accommodate the ROS-NETs functional axis for alleviating the rheumatoid lesions.

In the meantime, there are some limitations to proclaim in this study. Firstly, although neutrophils mostly activate in the early stage of RA, the late stage of the disease also involves other immune cells such as macrophages or T cells, which needs to be further unveiled in next study. Secondly, only RA is included in the present research, while other NETs-mediated diseases should be enumerated as well to provide more evidences for the application of HSI@ECM. Thirdly, concrete molecular mechanism between ROS scavenging and NETs suppression is still unclear in view of the current results, which might be the main investigative orientation in future.

## Conclusion

In summary, we developed a biomimetic nanocatalytic system (HSI@ECM) by coating HSI nanosheets with HUVEC-derived cell membranes. This bio-inspired design mimicked neutrophil chemotactic adhesion, enabling targeted delivery to activated neutrophils at inflamed joint sites. HSI@ECM efficiently scavenged excessive ROS, preserved mitochondrial function, and markedly suppressed NETs formation. In a CIA mouse model, HSI@ECM demonstrated superior therapeutic efficacy in alleviating joint swelling, soft tissue inflammation and cartilage-bone damage compared with both the uncoated HSI nanosheets and conventional drug MTX. Altogether, this study highlighted HSI@ECM as a promising nanocatalytic system for neutrophil-mediated inflammation and excessive NETs function, offering a targeting strategy for RA treatment.

## Supplementary Information

Supplementary Material 1

## Data Availability

The datasets used and/or analyzed during the current study are available from the corresponding author on reasonable request.
