# Supplementary material for: Nanocatalytic anti-inflammatory therapy for alleviating rheumatoid lesions with endothelial cell-derived hydrosilicene
Source: J Nanobiotechnology. 2026 Aug 15;24:1055. doi: 10.1186/s12951-026-04930-0 (PMC13753428; doi:10.1186/s12951-026-04930-0)
Supplement: Supplementary file 1 [file 12951_2026_4930_MOESM1_ESM.docx]

**Nanocatalytic anti-inflammatory therapy for alleviating rheumatoid lesions with endothelial cell-derived hydrosilicene**

Jize Dong3*, Weihan Yu3*, Moran Huang5*, Zhou Li3, Yeheng Lu1,2, Jiwu Chen3#, Zhixin Chen4#, Xingyu Zhang1,2#

1. Department of Plastic and Reconstructive Surgery, Shanghai Geriatric Medical Center, Shanghai 201104, China

2. Department of Plastic and Reconstructive Surgery, Zhongshan Hospital, Fudan University, Shanghai 200032, China

3. Department of Sports Medicine, Shanghai General Hospital, Shanghai Jiao Tong University School of Medicine, Shanghai 200080, China

4. Shanghai Frontiers Science Center of Nanocatalytic Medicine, Shanghai Tenth People’s Hospital, School of Medicine, Tongji University, Shanghai 200331, China

5. Department of Orthopaedics, Shanghai Sixth People's Hospital Affiliated to Shanghai Jiao Tong University School of Medicine, Shanghai 200233, China

* These authors contributed equally in this work.

\# Corresponding authors: Xingyu Zhang (zhang.xingyu@zsgmc.sh.cn), Zhixin Chen (chenzhixin19@mails.ucas.ac.cn), Jiwu Chen (jeevechen@gmail.com).

***Supplementary information***

Fig. S1. The real-time pictures of H_2_ gas generation upon adding the H_2_O_2_ solution.

Fig. S2. The cytotoxicity assay with the HUVECs of HSI nanosheets at different concentrations and time points (n = 3, ns represented no significant difference).

Fig. S3. The cytotoxicity assay with the HUVECs of HSI@ECM at different concentrations and time points (n = 3, ns represented no significant difference).

Fig. S4. The preparation and characterization of HSI@RBCM as a control. A) TEM image of the extracted RBC membranes (scale bar: 100 nm). B) SEM image of HSI@RBCM (scale bar: 200 nm).

Fig. S5. The isolation and characterization of human peripheral neutrophils by Wright-Giemsa staining (scale bar: 30 μm).

Fig. S6. The ROS scavenging efficiency curves for HSI nanosheets at gradient concentrations (n = 3).

Fig. S7. The ROS scavenging efficiency curves for HSI@ECM at gradient concentrations (n = 3).

Fig. S8. Analysis of the area under the curves shown in Fig. S6 and Fig. S7 for comparing overall ROS scavenging efficiency (n = 3).

Fig. S9. The representative immunofluorescence detection of PAD4 protein in control, PMA, HSI nanosheets, HSI@ECM and NAC groups (scale bars: 25 μm).

Fig. S10. The relative analysis of PAD4 protein expression according to the immunofluorescence results in Fig. S9 (n = 3, ***p < 0.001, ****p< 0.0001).
